# Validation of a digital food frequency questionnaire for the Northern Sweden Diet Database

**DOI:** 10.1186/s12937-024-00984-8

**Published:** 2024-07-24

**Authors:** Maria Wennberg, Lisa Kastenbom, Linda Eriksson, Anna Winkvist, Ingegerd Johansson

**Affiliations:** 1 Department of Public Health and Clinical Medicine/Sustainable Health, Umeå University, Umeå, S-901 87 Sweden; 2 Department of Odontology, Umeå University, Umeå, Sweden; 3 Department of Internal Medicine and Clinical Nutrition, The Sahlgrenska Academy, University of Gothenburg, Gothenburg, Sweden

**Keywords:** Food frequency questionnaire, FFQ2020, Northern Sweden Diet Database, Validity, Reproducibility

## Abstract

**Background:**

Dietary habits strongly influence health, with poor diets contributing to numerous deaths annually. Addressing this requires improved dietary habits and consistent monitoring thereof. In northern Sweden, a validated food frequency questionnaire (FFQ) has been used for decades, but trends show that its ability to accurately measure intake has diminished. With changing eating habits and food supply, updating the FFQ was crucial, leading to the development of FFQ2020. This study assessed FFQ2020’s relative validity using 24-hour recalls and evaluated its reproducibility.

**Methods:**

Participants were recruited from one of the northern-Sweden population-based health screenings and by advertising. Food intake was registered in an electronic food frequency questionnaire (FFQ2020) (test instrument) and reference data were obtained by six repeated electronic 24-hour dietary recalls (24HDR). Intakes of single foods were aggregated into food groups and healthy diet index scores, and daily energy and nutrient intakes were estimated. Results from the two methods were described and tested in univariate analyses and correlation tests, Bland Altman plots, cross-classification validity, and intra-class correlation analyses.

**Results:**

Totally, 628 adults were invited to participate in the study. Of these, 320 joined, and 244 completed at least four 24HDRs. The median intakes in food groups, as well as the mean index scores and estimated nutrient intakes, were largely similar between the FFQ2020 and 24HDR recordings. The correlation coefficients between the two assessments ranged from 0.253 to 0.693 for food groups, 0.520 to 0.614 for diet indices, and 0.340 to 0.629 for energy and nutrients. Intra-class correlation coefficients indicated at least good reproducibility for intakes of food groups, diet index scores, and nutrients. Generally, Bland-Altman plots did not reveal any gross systematic disagreement between the two methods for any of the assessments. However, there were single observations located outside the upper or lower 95% confidence interval (CI) limits for the difference between FFQ2020 and the 24HDR recordings.

**Conclusion:**

In concert, the results suggest that the relative validity and reproducibility of FFQ2020 are acceptable for trend analyses and group comparisons in large-scale studies but also that extended reference periods would improve the precision of less frequently consumed foods.

**Supplementary Information:**

The online version contains supplementary material available at 10.1186/s12937-024-00984-8.

## Background

An imbalanced diet, characterized by a low intake of fruit and vegetables and a high consumption of red meat, has been reported as the largest contributor to the overall disease burden worldwide, including 11 million deaths annually from dietary risks [1]. This calls for continuous monitoring of dietary habits in the population, as well as tracking of temporal trends in diet patterns, and assessing their associations with disease risks. Various instruments are available to assess dietary intake. The most used methods include food records, 24-hour dietary recalls (24HDR), and food frequency questionnaires (FFQs). While biomarkers can be valuable for monitoring intake of specific nutrients and metabolomics profiling for specific foods, they are less effective in capturing overall habitual dietary intake. All methods are, however, susceptible to both random and systematic biases, partly related to the proband and partly to the instrument itself [2]. Hence, it is crucial to gain insights into how well a specific instrument performs in a specific research context, within a specific population, and for an FFQ specifically ranking capacity. FFQs are comparably easy to administer, cost-effective, and less burdensome for the participants, making them a preferred instrument in large-scale epidemiological studies. However, data obtained from FFQs must be validated against a more accurate method before being utilized in research on diet and health.

The population of northern Sweden has since 1985 been part of two continuous population-based health screenings, the VIP (Västerbotten Intervention Programme) and the Northern Sweden MONICA (Monitoring Trends and Determinants in Cardiovascular Disease) screenings, which are part of the Northern Sweden Health and Disease Study (NSHDS). Diet monitoring is integrated into the protocol and the curated and quality-controlled dietary data make up the Northern Sweden Diet Database (NSDD). For dietary data collection, a self-administered FFQ was developed in 1984 and extensively validated [3–6]. However, over the past 38 years, significant changes have taken place in the variety of foods available in the market as well as in trends in food selection and portion sizes in the population [7, 8]. It is suspected that the FFQ used in the VIP and MONICA screenings has lost accuracy over time, as evidenced by a decrease in the recorded energy intake, despite an increase in the population’s mean body mass index, as reported for the VIP population by Winkvist et al. [9]. This loss of accuracy may be attributed to the absence of modern food items in the FFQ. To address this, we have developed a modernized, self-administered FFQ, named FFQ2020, which better captures contemporary dietary intake patterns. This includes plant-based replacements for meat and milk, newer varieties of pulses and grains, noodles, and additional dishes. Though the FFQ2020 was developed for the VIP and MONICA projects in Northern Sweden the design is suitable for any population-based study in Sweden. Temporal changes in dietary habits are reported for other countries too [10, 11] and hence possibly the FFQ2020 can be amended to serve populations outside Sweden.

Here, we present the relative validity of the FFQ2020 instrument (test instrument) in comparison with repeated 24HDR (reference). Additionally, we describe reproducibility based on repeated FFQ2020 assessments.

## Methods

### Study participants and study design

In the current study, where we aimed to estimate the relative validity and reproducibility of the FFQ2020, participants were recruited from the 8th screening of the Northern Sweden MONICA study, conducted in the spring of 2022. The MONICA study has invited population-based samples of 25–74-year-old inhabitants in Norrbotten and Västerbotten—the two northernmost counties of Sweden—approximately every fifth year since 1986 [12]. For our validation study, with data collection from January 2022 until September 2023, we specifically targeted MONICA participants aged 35–64 years to align with the age range of the VIP study. Invitations were sent via postal mail to those who in the 2022 MONICA survey had consented to be contacted for future studies. Additionally, we recruited participants in the same age group on a national level through advertisements. Of the 244 participants available for statistical analysis in this study 75.8% were recruited from the Northern Sweden MONICA study and 24.2% through advertisement.

### Dietary data collection

#### FFQ2020 (test instrument)

FFQ2020 is a modernized and updated version of the paper-version FFQ previously used in the Northern Sweden Health and Disease Study [3]. The FFQ2020, available from the Biobank Research Unit at Umeå University, Sweden (ebf@umu.se), is a semi-quantitative FFQ with 108 questions covering, e.g., fats, dairy products, bread and cereals, nuts and seeds, fruits and vegetables, potatoes, rice, pasta, noodles, legumes, grain products, meats, fish, eggs, and vegetarian options and drinks (see Table S1 for components covered by FFQ2020). Intake frequencies are reported on a nine-level scale; never, a couple of times a year, 1–3 times a month, once a week, 2–3 times a week, 4–6 times a week, once a day, 2–3 times a day or 4 times a day or more. Further, photos of four plates with increasing portion sizes are included for the participants to indicate their average portion size of (a) potato/ rice/ pasta/ noodles/ grains, (b) meat/ fish/ chicken/ vegetarian substitutes, and (c) vegetables (cooked or raw). Study data were collected and managed using the REDCap electronic data capture tools hosted at Umeå University, Sweden [13]. FFQ2020 data were collected between spring 2022 and spring 2023, and the respondents were asked to fill in their habitual consumption frequencies over the last year. For evaluation of reproducibility, all participants again filled out FFQ2020 7–12 months after entering the evaluation study. The baseline FFQ was used in the validation analyses, and the second FFQ for assessment of repeatability.

#### 24-hour Diet Recalls (24HDR, reference instrument)

Data from repeated 24-hour recalls (24HDR) collected through the web-based dietary assessment tool RiksmatenFlex, the Swedish Food Agency (https://www.livsmedelsverket.se), were used as reference method. RiksmatenFlex has been validated through recall interviews in a national dietary survey of adolescents [14], where the estimated energy and food intake from RiksmatenFlex were found to be comparable to those from the recall interviews. The participants were contacted by e-mail with a request to enter everything they ate or drank during the previous 24 h, including amounts, in the online system. This was performed for six randomly selected days (including weekdays and weekend days) over a period of around ten weeks and introduced 3–6 months after the baseline FFQ was completed.

### Background data

Relevant background data for the calculation of energy needs and data relevant for the comparison of groups were asked for in the screening questionnaire. These included year of birth, sex, educational level (four levels from not completed primary school to university), physical activity (how often did you exercise the last three months with four options from never to more than 3 times a week), and smoking habits (do you smoke cigarettes with options on never, past or present use). Height and weight were measured at the screening visit.

### Dietary data processing

First, reported intake frequencies of the 108 food/food-aggregated questions in FFQ2020 were transformed to intakes per day (0 to 4 intakes/day). To estimate the corresponding daily intake frequencies from the 24HDR recordings, all registered foods were classified according to the content of the 108 FFQ questions, aggregated within each group and participant, and divided by the number of completed 24HDR days. The numbers of intakes per day were aggregated to estimated intakes in 30 food groups used either for the validity analyses per se, or to calculate scores for three a priori diet pattern indices (relative Mediterranean Diet Index, rMED [15], Healthy Nordic Food Index, HNFI [16], and Plant-based Diet Index, PDI [17]), and to evaluate data-driven diet patterns from the test and reference recordings (Table S1). The characteristics of the three diet pattern indices are presented in Table S2.

Daily energy and nutrient intakes were estimated by weighting the daily intake frequencies by amounts eaten (portion) and energy/nutrient content in the Swedish Food Composition Database at the Swedish Food Agency (data retrieved 1 July 2023). The portion sizes were either estimated from the portion size indication on the photos, by average natural sizes of foods, e.g., an egg or an apple, or from sex and 10-year age group median intakes from recordings from the Riksmaten Adult project (downloadable at https://www.livsmedelsverket.se/om-oss/psidata/apimatvanor [18]), after controlling that the levels were largely in line with those reported in the 24HDR recordings.

Diet data in NSDD are quality defined at three levels, i.e., having left one of the portion indications unanswered (exclude level 2), having indicated portion sizes but left ≥10% missing FFQ questions (exclude level 1), and meeting none of these criteria (exclude level 0, i.e., to be kept for analyses). To further identify potential inaccurate dietary recordings, daily energy intake was compared to sex, height, body weight, and age-relevant resting energy expenditure requirements, i.e., need at a resting physical activity level (PAL) [19]. A PAL value below 0.7 (corresponding to the 5% lowest values from the PAL distribution here) was considered severe under-reporting and above 3.0 as an implausible over-reporting. Though all 244 study participants fulfilled the basic NSDD quality criteria, i.e., exclude level 0, 13 participants displayed very low PAL-values (5% with < 0.7) or high (1% with ≥3.0) based on the FFQ2020 or 24HDR recordings (Figure S1). To adhere to the present recommendation when using NSDD data, i.e., to exclude participants with the lowest and highest relative energy intakes, we report data for all 244 participants with four or more 24HDR but also present results where those with low/high PAL are excluded or indicated.

### Statistical analyses

Descriptive statistics for the intakes in food groups are presented as medians and 25 and 75% percentile limits. Associations between food group estimates from the test and reference methods were assessed by Spearman correlation coefficients. As ranking-based assessments are common in nutritional epidemiological studies, the participants were classified into tertile groups by recording method and cross-tabulated to assess the agreement between classifications by the FFQ2020 versus that by the 24HDRs.

Descriptive statistics for diet index scores, estimated energy, and absolute nutrient intakes are presented as mean values with standard deviations (sd). Here, associations between estimates from the test and reference methods were assessed by Pearson correlation coefficients but Spearman correlations are also shown. To mitigate the effects of measurement error in diet data collection, it is common to energy-adjust nutrient intakes. Therefore, the analyses were repeated for energy-adjusted nutrient estimates using (a) the residual method, i.e., from a regression model with total energy intake as the independent variable and absolute nutrient intake as the dependent variable with the residuals representing an energy-independent estimate of nutrient intake [20], or (b) the energy density method, i.e., units per 1,000 kCal.

Intra-class correlations (ICC) were calculated to estimate the reproducibility of FFQ2020 measurements, i.e., intra-rater class correlations between the two recordings using a 2-way mixed-effects model with an agreement coefficient and average measures. ICC-values below 0.5 were considered as poor reproducibility, between 0.5 and 0.75 as moderate, between 0.75 and 0.9 as good, and a value above 0.9 as excellent reproducibility [21].

Bland-Altman analyses were used to indicate systematic disagreement between measurements from the test and reference methods as well as among the repeated FFQ2020 measurements [22]. These results are shown in Bland-Altman plots with the 95% CI of the mean difference indicated. For values within the 95% CI, no significant systematic bias was inferred whereas a skewed distribution outside the 95% CI indicated a systematic bias.

Data-driven analyses are increasingly utilized in the field of nutritional epidemiology. However, there are limited reports on the accuracy of using FFQ data in such analyses. We assessed this by comparing loading plots from Principal Component Analysis (PCA) models based on dietary recording information obtained by the test method and a reference method, respectively. The analysis was conducted using SIMCA 17 software (Sartorius Stedim Biotech, Umeå, Sweden). The data in the PCA models were auto-transformed as per SIMCA’s rules, which included logarithmic transformation and auto-scaling to unit variance. This process ensured that all variables were given equal weight. The outcomes of the models are presented in terms of model explanatory values (R²) and cross-validated predictive values (Q²). For cross-validation, every seventh observation was omitted, and its value was predicted using models built from the remaining observations.

## Results

### Drop-out analysis and dietary recording quality

**Fig. 1:**
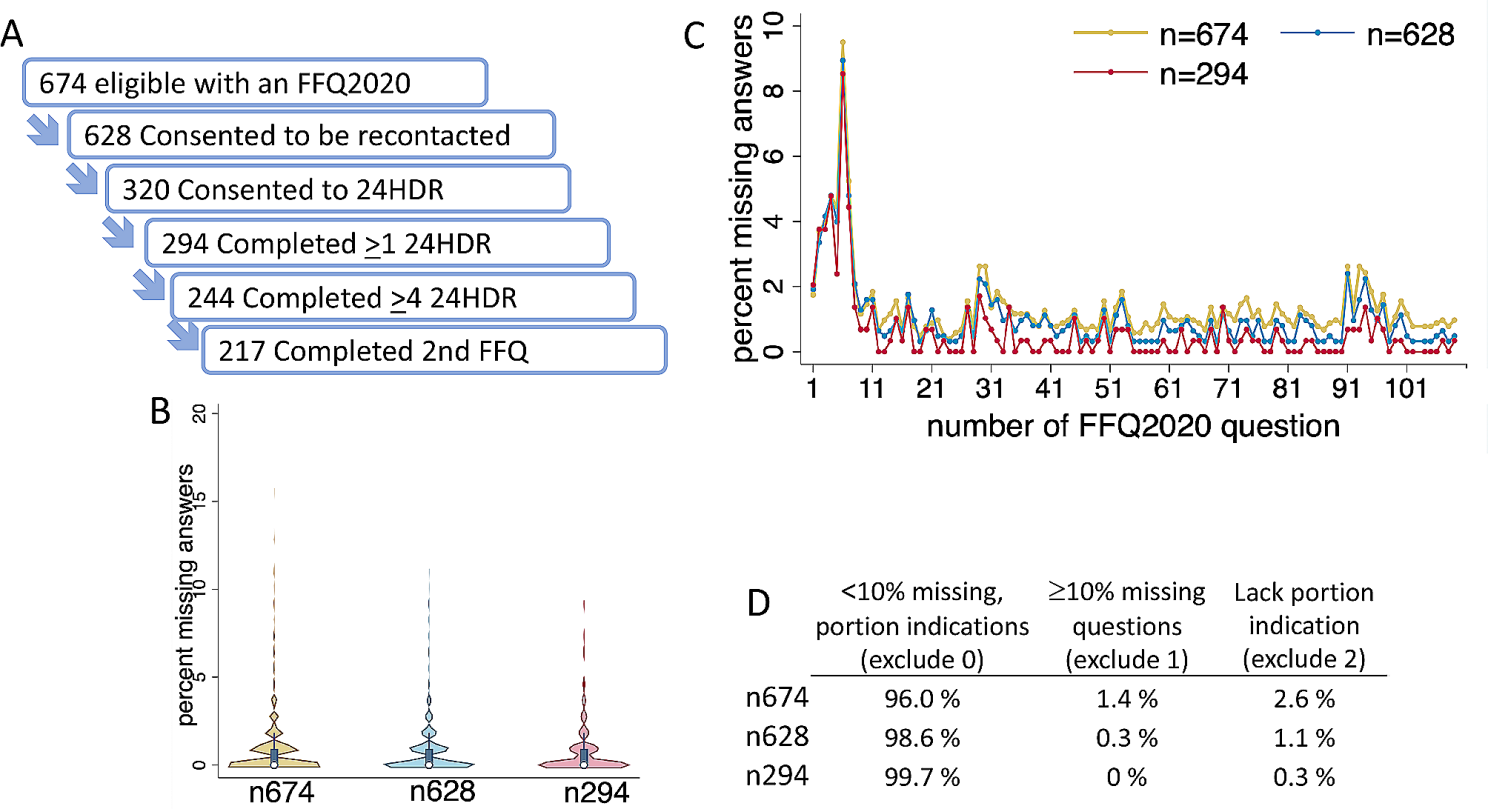
Flow of participants and recording quality. (**A**) participant flow chart; (**B**) proportion (%) missing answers across study participants, (**C**) across the 108 FFQ2020 questions, and (**D**) classification by a three-tiered level exclusion score used in NSDD where participants in exclude level 0 have < 10% missing answers and complete portion size indications, in exclude level 1 have ≥10% missing answers and complete portion size indications, and those in exclude level 2 lack ≥1 portion size indication

A total of 674 eligible persons had completed a baseline food frequency questionnaire (FFQ2020). Of these persons, 93% (*n* = 628) had agreed to and were recontacted for participation in the present validation study, which included repeated 24-hour dietary recalls (24HDRs) and a follow-up FFQ2020. When invited to begin the 24HDR recordings, 320 participants provided their consent. Out of these, 294 completed at least one 24HDR, and 244 completed four or more 24HDRs (Fig. 1A). This latter group constituted the validation group, serving to estimate the relative validity of FFQ2020 recordings (the test method) in comparison to repeated 24HDR recordings (the reference method). A drop-out analysis was done for the remaining 430 participants who only completed the baseline FFQ2020 (the drop-out group).

In both the validation and drop-out groups, a higher proportion of women than men was observed (Table 1). Participants in the validation group were more likely to have a university education and appeared to lead healthier lifestyles, as suggested by a lower mean body mass index (BMI) and higher levels of physical activity, compared to those who dropped out. The percentage of participants who reported that they had never smoked was similar in both groups.

**Table 1:** Characteristics of the validation and drop-out groups

	Validation group	Drop-out group
Number	244	430
Proportion men/women	38.9/61.1	41.9/58.1
Age, years, mean (sd)	49.8 (8.5)	50.3 (8.7)
BMI, mean (sd)^1^	26.1 (4.3)	27.6 (5.6)
Never smoker, %	75.0	71.4
Education, % with university level	61.5	46.1
Physical activity, % ≥twice weekly	43.8	29.1
Proportion with “optimal” FFQ2020 (exclusion score 0)^2^	100	96.0

^1^Mean BMI (body mass index) was adjusted for sex and age in general linear modeling (glm) and the *p*-value between the two groups was < 0.001

^2^Complete portion indications and ≤10% missing FFQ2020 questions

In the baseline FFQ2020 survey, 87% of participants had two or fewer missing responses out of the 108 questions. This trend of minimal unanswered questions was consistent across the eligible participants (n = 674), those who consented to participate (n = 628), and those who completed at least one 24-hour dietary recall day (24HDR) (n = 294) (Fig. 1B). Further, less than 2% of responses were missing across the 108 food-questions, except for three specific questions regarding the type of fat used on bread (Fig. 1C). Furthermore, the FFQ2020 data from 98% of the eligible participants and 100% of those who completed at least one 24HDR were categorized as ‘exclude level 0,’ indicating ‘good’ quality data (Fig. 1D).

### Intakes of 30 food groups estimated by the test and reference methods

**Fig. 2:**
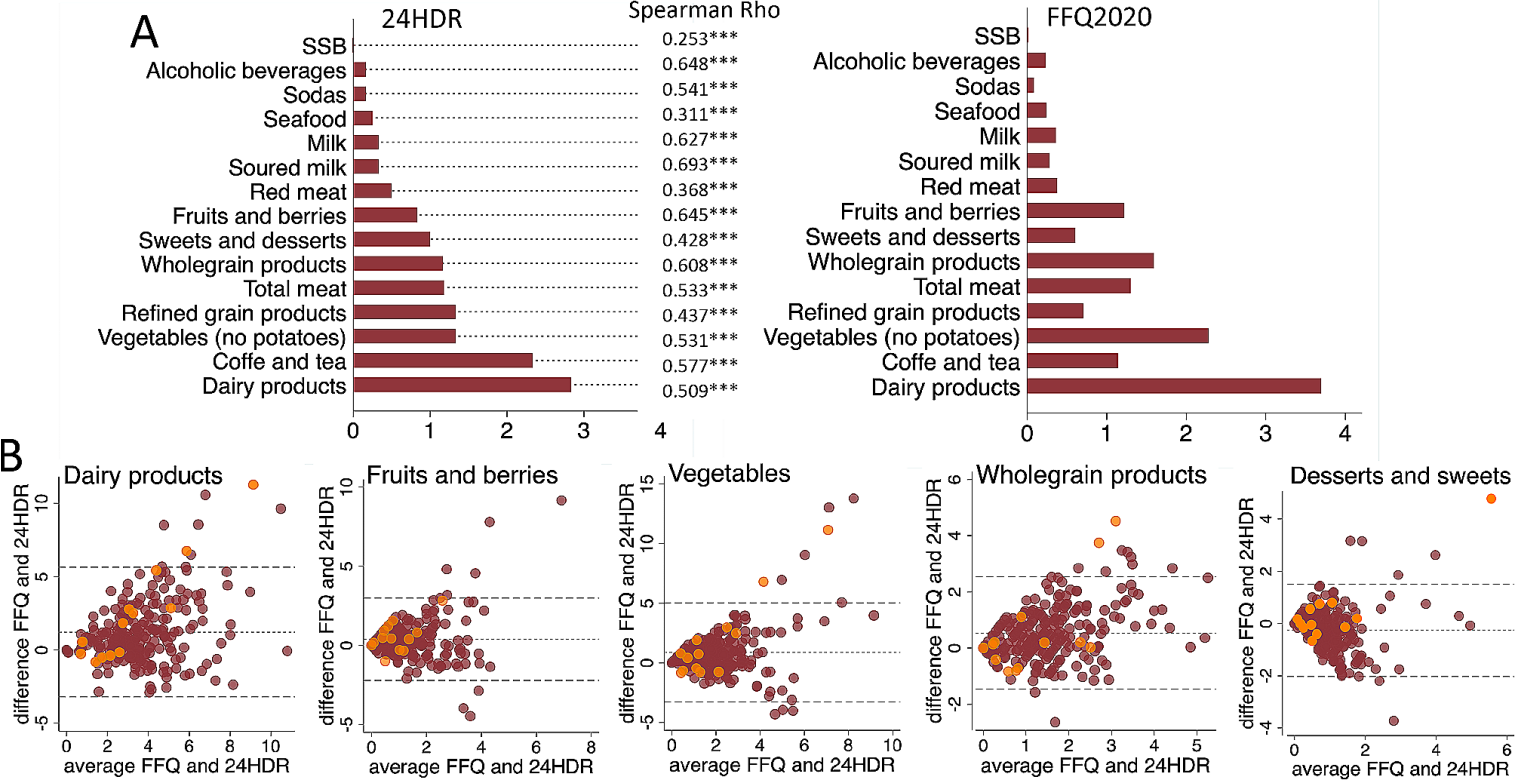
Relative validity of FFQ2020 food group assessments. (**A**) Median daily intakes in food groups by 24HDR and FFQ2020, respectively, with Spearman correlation coefficients; (**B**) Bland Altman plots illustrating agreement between the two methods for dairy products, fruits and berries, vegetables (without potatoes), wholegrain products, and desserts and sweets. The plots incorporate data from all 244 participants, and the 13 participants with extreme PAL values are highlighted in orange. *** for *p* < 0.001

The relative validity of intakes across 30 food groups (Table S1) was assessed among the 244 study participants who completed at least four 24HDRs. Of these, median intakes of 15 foods/food groups frequently examined in diet-health association studies are displayed in Fig. 2A. Data for all 30 groups, including medians, and 25 and 75 percentile limits are found in Table S3. In general, the FFQ2020 and the repeated 24HDRs yielded similar trends in median food group intakes. However, the FFQ2020 tended to yield higher intake estimates for some food groups (e.g., dairy products) and lower for others (e.g., refined grain products), compared to the 24HDRs (Fig. 2A, Table S3). Spearman correlation coefficients ranged from 0.693 (soured milk products) to 0.253 (sugar-sweetened beverages) (Fig. 2A, Table S3). Strong correlations (coefficients≥0.60) were found for alcoholic beverages, milk, soured milk products, fruits and berries, wholegrain products, and coffee. Moderate correlations (coefficients ≥0.40 to < 0.60) were noted for the other food groups, apart from sugar-sweetened beverages, seafood, sausages, red meat, and potatoes (Fig. 2A, Table S3). Bland-Altman plots for five food groups commonly evaluated in studies on diet and health, i.e., dairy products, fruits and berries, vegetables, wholegrain products, and desserts and sweets, are shown in Fig. 2B. The plots incorporate data from all 244 participants, but the 13 participants with extreme PAL values are highlighted in orange. The plots did not reveal any gross systematic differences between the two dietary assessment methods. This conclusion is based on the fact that observations falling outside the 95% CI limits were predominantly evenly distributed below and above these limits (Fig. 2B).

### Cross-classification by intakes of 30 food groups estimated by the test and reference methods

Tertile cross-classifications of food group intakes based on ranking by the test and reference methods for the 244 participants are shown in Table S4. There were marked variations among food groups regarding the proportion of participants who were correctly classified into the highest or lowest tertile by both methods. On average, 62% of the study participants were correctly allocated to the highest tertile by both methods and 53% to the lowest tertile. For food groups that were reported at least once in the 24HDR recordings, a correct group allocation into the lowest tertiles varied from 81% for coffee to 37% for potatoes. The corresponding numbers for the highest tertile were 79% for soured milk products and 39% for poultry. Less than 22% were grossly misclassified for the highest FFQ tertile, i.e., were not in the highest or adjacent 24HDR tertile.

### Food intake patterns and their cross-classification estimated by the test and reference methods

**Fig. 3:**
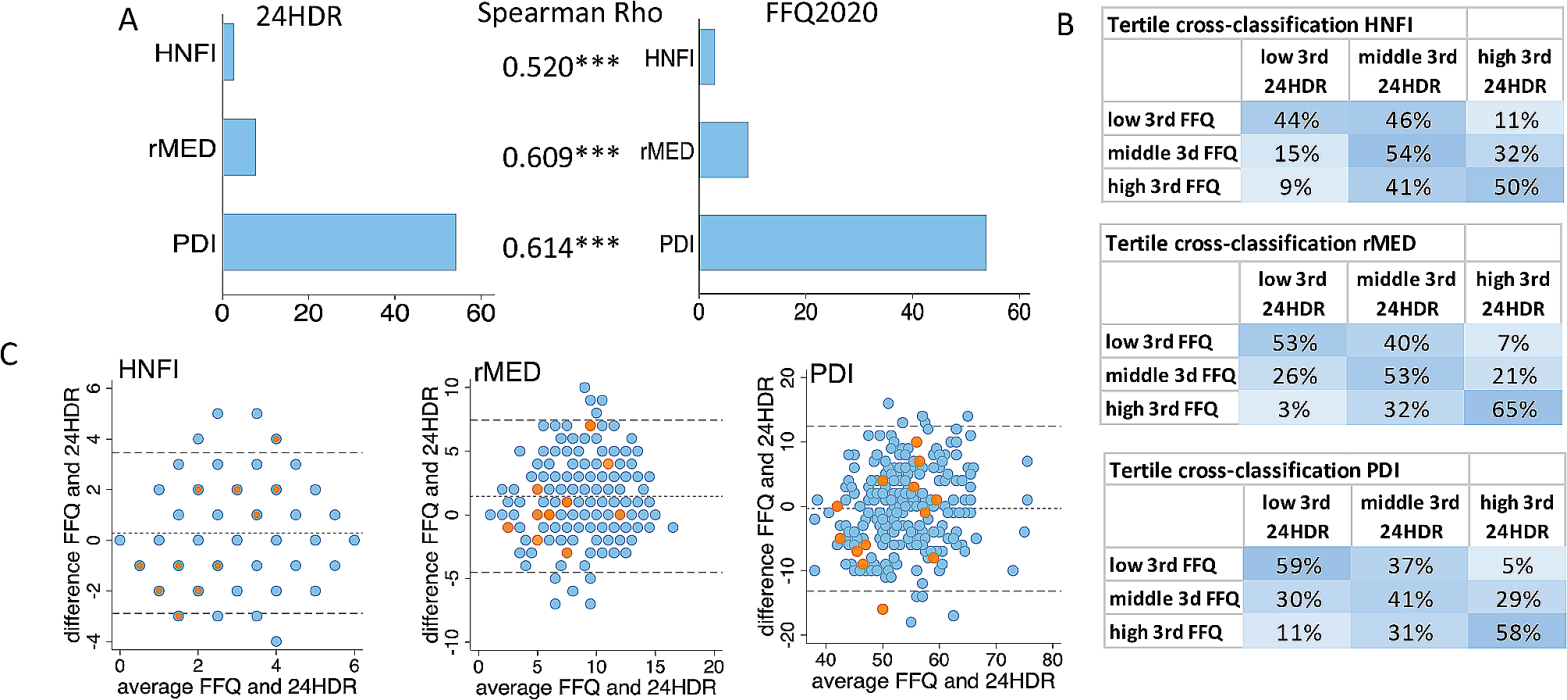
Relative validity of the Healthy Nordic Food Index (HNFI), relative Mediterranean Diet Index (rMED), and the Plant-based Diet Index (PDI). (**A**) Mean score and correlation between index scores assessed from 24HDR and FFQ2020 recordings. (**B**) Tertile cross-classifications for the three indices by test and reference estimations (**C**) Bland Altman plots for agreement between the two methods. The plots incorporate data from all 244 participants, and the 13 participants with extreme PAL values are highlighted in orange. *** for *p* < 0.001

The scores for the a priori diet pattern indices HNFI, rMed, and PDI were compared based on the test and reference methods. The two methods yielded similar mean scores for all three indices (Fig. 3A), with correlation coefficients ranging from 0.520 (HNFI) to 0.614 (PDI) (Fig. 3A). Between 65% (rMED) to 44% (HNFI) were correctly classified into the highest or lowest tertile by both methods (Fig. 3B). The Bland Altman plots did not reveal any systematic deviations between the recording methods (Fig. 3C).

**Fig. 4:**
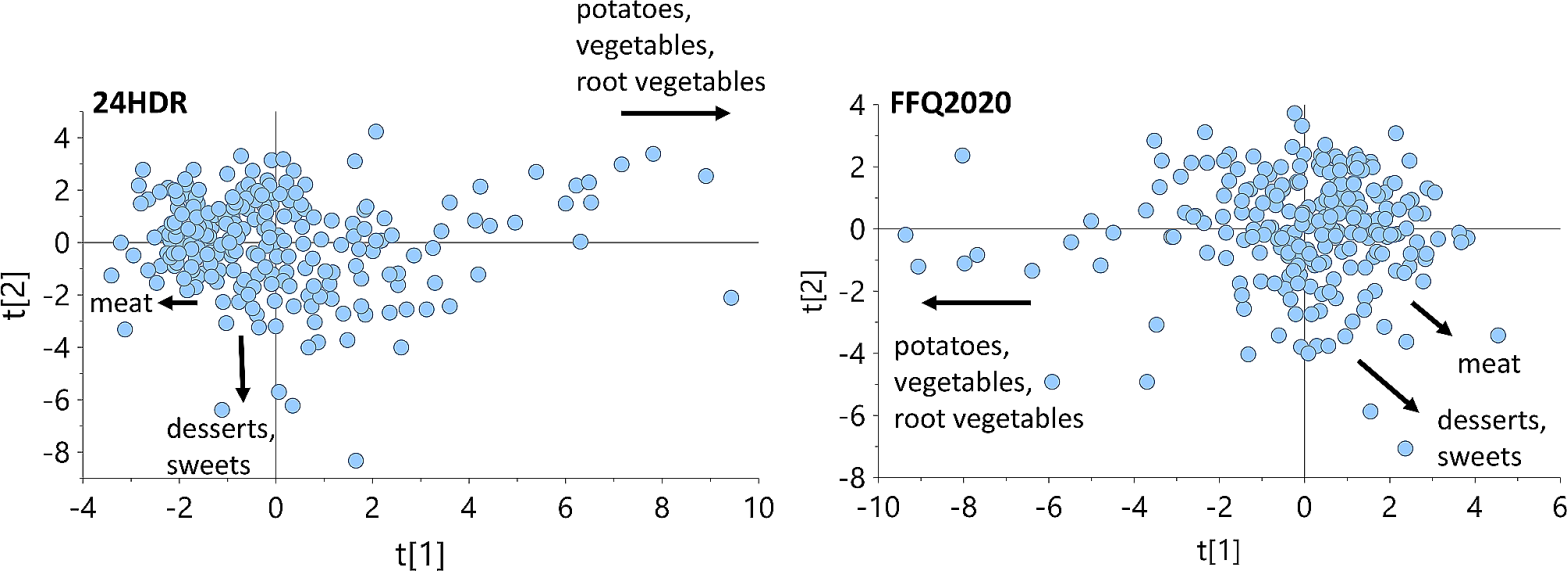
Dietary patterns from PCA clustering. Score plots from (**A**) the 24HDR recordings and (**B**) the FFQ2020 recordings

Further, the same food groups from the test and reference methods were major determinants for the eating patterns in data-driven modeling (PCA) using the 30 food groups presented in Table S1. The PCA models had moderately strong explanatory power (R^2^), i.e., 27% of the variation was explained by the food group recordings in the test method and 29% in the reference method. The corresponding cross-validated prediction values (Q^2^) were 10% and 17%, respectively. The PCA score plots identified three food groups as the most influential for eating patterns, regardless of the recording method: (i) potatoes/vegetables/root vegetables, (ii) meats, and (iii) desserts/sweets (Fig. 4).

### Estimated energy and nutrient intakes by the test and reference methods

**Fig. 5:**
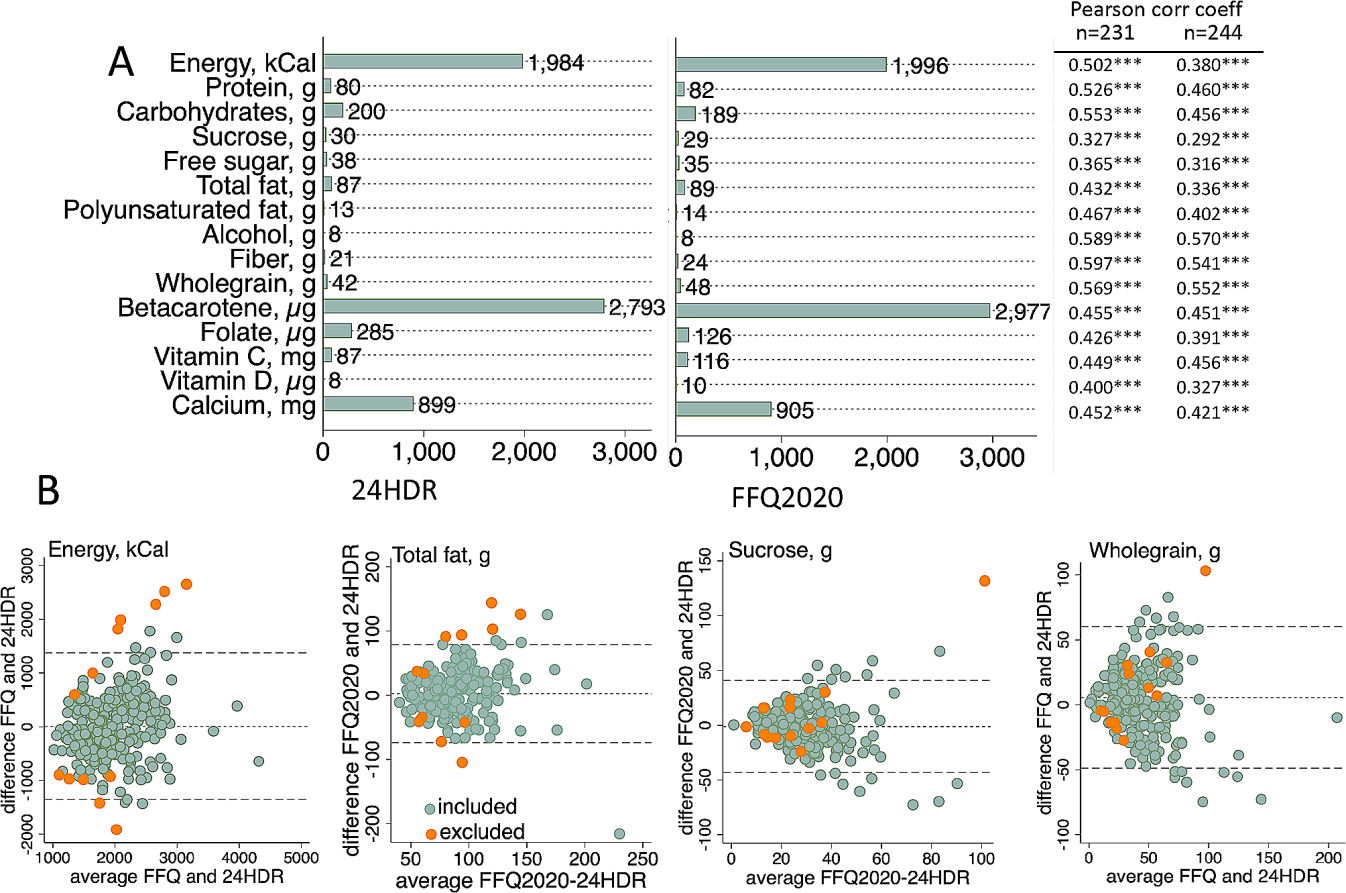
Validity of FFQ2020 energy and nutrient assessments. (**A**) Reported daily mean intakes by 24HDRs and FFQ2020, respectively (*n* = 244), and correlation coefficients for absolute values for the 244 and 231 participants, respectively; (**B**) Bland Altman plots for agreement between the two methods for energy (kCal/day), total fat (g/day), sucrose (g/day), and wholegrain (g/day). The plots incorporate data from all 244 participants. The 13 participants with extreme PAL values are highlighted in orange. *** for *p* < 0.001

Further, the relative validities for the estimated intake of energy and 14 nutrients were evaluated (Fig. 5, Table S5, Table S6). The mean intakes of energy and the evaluated nutrients were highly similar for FFQ2020 and the 24HDRs recordings among the 244 participants (Fig. 5A) and after exclusion of the 13 participants outside the defined PAL limits, as well as with or without adjustment for estimated energy intake (Table S5). However, the correlation coefficients between estimates from the two methods were generally lower in the group without any exclusions (*n* = 244) compared to the group with PAL-based exclusions (*n* = 231) (Fig. 5, Table S5). For the group with PAL exclusions, correlation coefficients for energy and macronutrients indicated a moderate agreement (Pearson or Spearman coefficients≥0.33 to < 0.55). Bland Altman plots did not indicate any systematic deviation between the two recording methods for energy and macronutrients if the evaluation was limited to the 231 participants with a PAL value within the defined limits as shown for energy and total fat in Fig. 5B. In contrast, the exclusion of the 13 participants outside the PAL limits did not affect the pattern for sucrose and wholegrains (Fig. 5B) or other non-energy nutrients (Table S5).

Cross-classifications of the participants into tertile groups based on estimated energy and nutrient distributions were done for both the 244 and 231 participants (Table S6). On average, 53% of the participants (*n* = 244) were correctly allocated to the highest or the lowest tertile based on absolute intake. The corresponding number was 55% for absolute intake among the 231 participants. Generally, energy adjustment slightly increased the proportions correctly classified.

### Reproducibility of FFQ2020-derived information

**Fig. 6:**
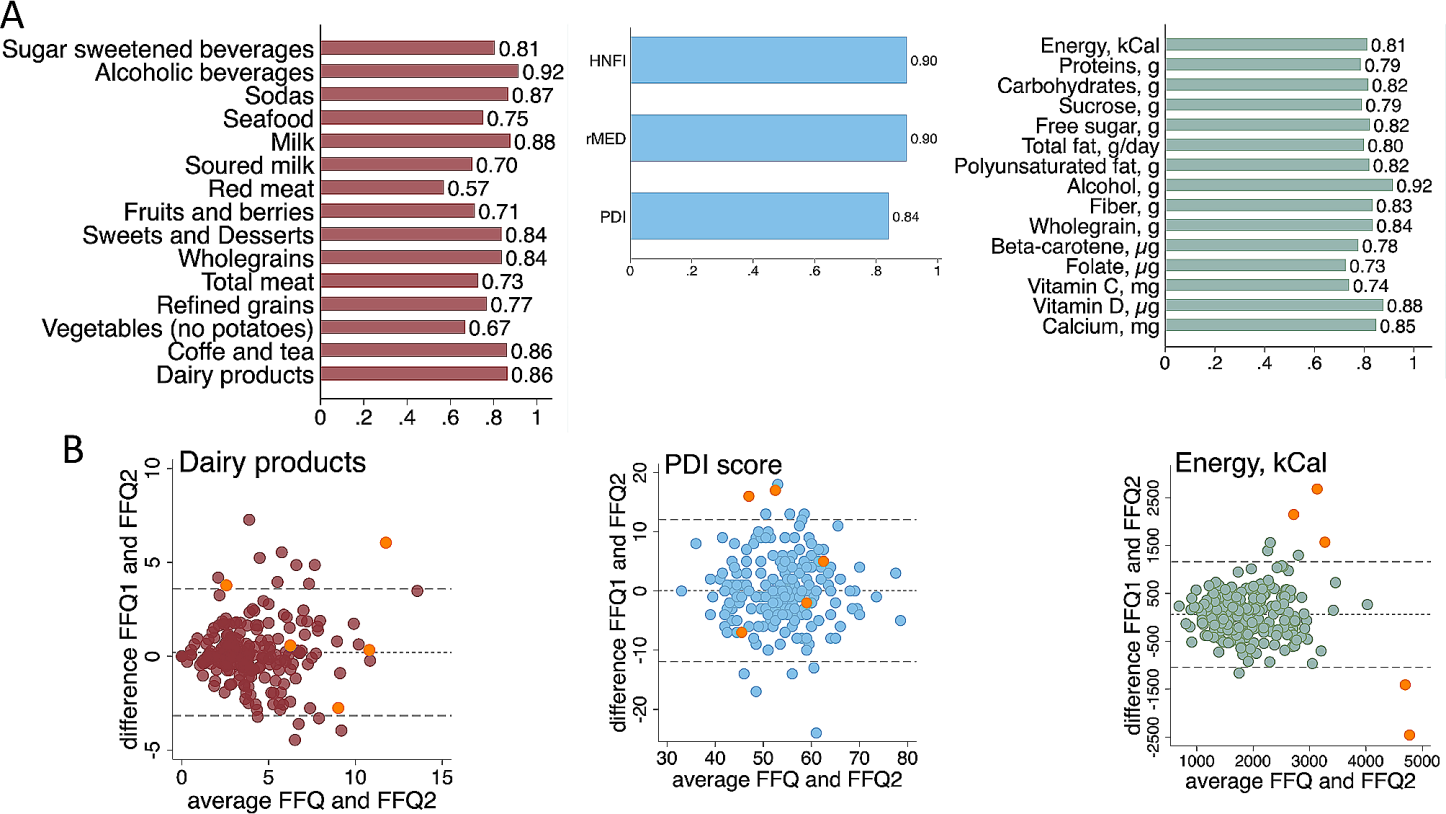
Reproducibility of FFQ2020 data. Bar graphs showing (**A**) intra-class correlation coefficients for food groups, diet indices, and energy and 14 nutrients, and (**B**) Bland Altman plots for one representative item for the food groups, diet indices, and energy. The plots incorporate data from all 217 participants, and the participants with extreme PAL values are highlighted in orange

Finally, also reproducibility of FFQ2020 was evaluated. Among the 244 participants who had recorded at least four 24HDR days, 217 respondents completed a second FFQ2020. The ICC correlation for food groups, diet indices, and nutrients were all moderate or higher, with the majority being good and some being excellent (Fig. 6A, Tables S7, Table S8). The Bland Altman plots did not reveal any major systematic imbalance, as shown here for dairy products, the PDI index, and energy (Fig. 6B), but the exclusion of participants with a PAL value outside the defined limits improved the agreement for energy and macronutrients.

### Sensitivity analyses in gender strata

Gender-stratified Spearman correlations between intakes based on FFQ and 24HDR recordings were run for the 30 food groups and three diet indices (Table S9). For both men and women, the correlation coefficient followed those reported for all participants (Spearman correlation 0.898 and 0.913, respectively). Though there were no major differences between men and women for the correlation levels for single food groups or the diet indices, women generally had slightly higher correlations (Table S9).

## Discussion

The present study evaluated the relative validity and reproducibility of the assessment of foods, food intake patterns, energy, and nutrient intakes by a digital FFQ (FFQ2020) constructed to reflect today’s food supply in Sweden and analogous countries. These analyses revealed the relative validity of intake in most food groups and some nutrients as high and that of energy and most other nutrients as acceptable. Correlation, Bland Altman plots, and ICC analyses revealed a high reproducibility of intakes in food groups as well as of estimated energy and nutrient intakes. Further, the FFQ2020 seemed user-friendly as indicated by the low proportion of missing answers and the very high proportion judged as exclude level 0, i.e., eligible for data inferences. Taken together, the results indicate that the FFQ2020 is suitable for large-scale studies on diet and e.g., health outcomes.

FFQ2020 was developed in response to the noted reduction over time in reported energy intakes concurrent with increasing BMI in the Västerbotten population [9], when a paper-based FFQ developed in 1984 was continually used. A reason for keeping the same instrument over the years has been to allow comparisons over time of consumption patterns (see e.g., Johansson et al. 2012 [7]). Still, for an FFQ to be valid and informative it must capture the core intake patterns of the population, and it was time for a more modern FFQ covering contemporary food items.

The relative validity of FFQ2020 was evaluated using four or more repeated 24HDR as the reference method. Spearman correlation coefficients ranged for food groups from 0.253 (sugar-sweetened beverages) to 0.693 (soured milk products), for a priori diet pattern indices between 0.520 (HNFI) to 0.614 (PDI), and for energy and unadjusted nutrient intakes between 0.340 (vitamin D) to 0.629 (alcohol). For the macronutrients, all correlation coefficients were in the range of moderate agreement. In data-driven modelling, the same food groups were major determinants for the eating patterns discovered based on input data from both methods. Finally, cross-classification for energy and nutrients showed on average that 55% were correctly categorized in the lowest and highest tertile, respectively, (absolute intake data, *n* = 231), and corresponding numbers for energy-adjusted intake data were close to the same.

Several studies have been published recently that also evaluate the relative validity of an FFQ and that used similar metrics and were performed in areas of relative geographical/ cultural proximity to our study. Researchers at Harvard have evaluated the relative validity of their updated FFQ long used in the US Nurses’ Health Study and Health Professionals Follow-up Study [23]. Here, the 149-food item and 25-food group item FFQ was compared with two 7-day food records conducted 6 months apart. Like in our study, participants with unreasonably low or high energy intakes were excluded from analyses. Spearman correlation coefficients for foods were on average 0.41 among women and 0.45 among men; values that are in similar ranges to those identified in our study. The Harvard study used an FFQ with larger number of food items than we did, and the reference method used (two 7-day food records) was more detailed and provided higher precision than did our reference method (repeated 24HDR). Even so, the correlation coefficients found in both studies were in similar ranges. This may reflect the fact that intake patterns captured by a retrospective method like FFQ differ from intake patterns captured by prospective methods such as food diaries and 24HDR. Therefore, while dietary assessments using food diaries/24-hour dietary recalls, or food frequency questionnaires demonstrate high precision, expecting correlations higher than those reported in both our study and the referenced Harvard study may be unrealistic, given the comparison between retrospective and prospective dietary intake. In the Gu et al. study [23], beverages had the highest while eggs and meat had the lowest correlation between the test and reference methods; again similar to the results in our study. Further, like in our study the lowest values were found for sugar-sweetened beverages and highest for alcohol. This likely reflects that both retrospective and prospective methods capture food items consumed often and regularly better than food items consumed occasionally and irregularly. Gu et al. observed higher correlation levels for food groups compared to individual food items, which aligns with our findings from Bland-Altman plots where single food items demonstrated less agreement than larger food groups. We suggest that this reflects the broader range and greater variation observed when groups are formed. Also, larger food groups likely are consumed more often and regularly than are each single food item, again yielding better agreement between retrospective and prospective methods. Furthermore, the use of food groups involves fewer transformation steps and introduces fewer errors, offering an advantage over nutrients in studies. The same researchers also evaluated the relative validity of their FFQ against 4 repeated 24HDR among women [24, 25] and men [26]. Energy-adjusted Spearman correlation coefficients were on average among women 0.43, ranging from 0.23 for lycopene to 0.75 for alcohol, and among men 0.52, ranging from 0.29 for lauric acid to 0.75 for coffee consumption. Correlations were similar across strata of BMI, age, and source of participants, which is of interest when judging our results because we were unable to conduct such stratified analyses due to limited power.

In another validation study performed in the US, a 63-item FFQ was compared with six or more 24HDR [27]. Energy-adjusted Spearman correlation coefficients were 0.50 and 0.52 among men and women, respectively (range 0.05–0.82), thus correlations were similar for both sexes similar to what we found for FFQ2020 for food groups and a priori diet indices. Their coefficients were highest for beverages and lowest for pasta and regular-fat yoghurt, thus partially similar to our results. Values were higher for a diet quality score than for food groups (0.69 and 0.61 for men and women, respectively). Also, the authors reported correlations to be higher for broader food groups (e.g., fruits) than for more specific and narrow food groups (e.g., citrus fruits), again in agreement with our results. As mentioned above, this likely reflects that larger groups of combined food items yield better agreement than do smaller units when different methods are compared. Overall, 25 of the 63 food groups had > 80% classified in the same or adjacent quartile based on both methods.

In Norway, researchers have compared a 279-item FFQ with three 24HDR [28] with respect to nutrients and foods. Spearman correlation coefficients ranged between 0.19 (iodine) to 0.69 (vitamin D) for nutrients and between 0.31 (fatty fish) to 0.71 (juice) for foods. In cross-classification, highest agreement was found for fruits and berries and vitamin D, and lowest agreement was found for meat, blood and offal. In Denmark, a 376-item FFQ was compared with three 24HDR [29]. Energy-adjusted Spearman correlation coefficients ranged from 0.18 to 0.58 for energy and nutrient intakes, and 28–47% of participants were classified into the same quartile based on both methods. Comparisons can also be made with a study from Cyprus [30]. Here, a 171-item FFQ was compared with three 24HDR with respect to nutrients. Spearman correlation coefficients ranged between 0 (iron) to 0.49 (magnesium). These studies thus exhibited broader ranges of agreement than did our study.

Similar validation studies also have been performed in populations with dietary habits less similar to those in Sweden. Imaeda et al. (2021) conducted a relative validation of a 47-item FFQ against four 3-d dietary record in a Japanese population [31]. For 20 food groups, energy-adjusted Spearman correlation coefficients were among men 0.44 with a range of 0.11–0.71, and among women 0.39 with a range of 0.17–0.72. Proportion of cross-classification into the same or adjacent intake quintile was 58–86% among men and 57–86% among women. Again, these results are in the same ranges as those of our study. In another Japanese study, a food combination questionnaire was compared with four dietary records with respect to nutrients, foods, and overall diet quality [32]. Median Spearman correlation coefficient for 16 food groups was 0.32 among women and 0.38 among men, and median Pearson correlation coefficient for 46 nutrients was 0.34 among women and 0.31 among men. For the diet quality indices, correlation coefficients ranged from 0.37 to 0.46. A small relative validation study conducted among 49 Chinese participants compared a 79-item FFQ with three 24HDR [33]. For 18 food groups, the average Spearman correlation coefficient was 0.27. Finally, in Nigeria a relative validation of an FFQ was recently conducted [34]. Here, a 60-item FFQ was compared with three 24HDR. Unadjusted Spearman correlation coefficients ranged between − 0.06 (smoked beef/goat) to 0.55 (smoked fish and soft drinks). For macronutrients, the values ranged between 0.14 (fat and fiber) to 0.63 (carbohydrates). The proportion of participants classified into the same intake quartile ranged from 25.6% (fat) to 34.9% (carbohydrates). In sum, the validity of our FFQ2020 seems to be on par with or even slightly higher than that of several other recently evaluated FFQs, in general values seem to be higher for larger food groups than for single food items and highest for beverages in most studies, and few differences have been found in relation to sex, BMI or age in the other studies. Take-home messages for future validation studies are to have reasonable expectations on correlation coefficients when retrospective and prospective methods are compared, that agreements will be higher for items consumed more often and more regularly, and for larger composite scores/groups than for single food items. Hence, these aspects should all be covered by both methods, whereas participant characteristics such as sex, BMI, or age are less important to control.

For the 217 participants who filled out FFQ2020 one more time 7–12 months after the first time, ICC correlations for the most common food groups ranged from 0.57 (red meat) to 0.92 (alcoholic beverages), and ICC correlations for nutrients from 0.73 (folate) to 0.92 (alcohol). Gu et al. [23] found an average ICC of 0.64 for their evaluated foods, ranging from 0.37 for fat-free cookies/brownies among men to 0.89 for liquor among men. Beverages had also here the highest reproducibility, whereas eggs and meat had the lowest. ICC values were higher for food groups than for individual foods (on average 0.71 for women and 0.72 for men). ICCs for energy and nutrients had among women a mean of 0.68 with a range of 0.50–0.91, and among men a mean of 0.69 with a range of 0.49–0.89 [24, 26]. In the other US study [27], ICC was > 0.50 for 83–97% of their 63 food groups for different strata on sex and race. In the Norwegian study, ICC ranged between 0.10 and 0.81 for nutrients and foods [28]. In Denmark, Rostgaard-Hansen and colleagues (2023) reported ICC between 0.52 and 0.88 for energy, nutrients, and foods [29]. The validation study in Cyprus obtained a median ICC of 0.46 (range 0.38–0.52) for nutrients [30]. In Israel, researchers compared a 133-item FFQ with three 24HDR [35] to evaluate reproducibility. That study obtained ICC between 0.55 and 0.83 for nutrients and between 0.58 and 0.79 for food groups. In the study in Japan, Imaeda et al. (2021) obtained ICC for 20 food groups of on average 0.61 among men with a range of 0.38–0.86, and among women of 0.66 with a range of 0.45–0.84 [31]. Finally, in the Chinese study [33], average ICC was 0.35 for the 18 food groups. In sum, our FFQ has a reproducibility in line with or even higher than other recently evaluated FFQs.

The number of missing answers in FFQ2020 was generally low, with 87% of the participants having only two or fewer missing responses. For three questions, concerning type of fat on bread, there was a higher proportion of missing answers. This is likely due to one type of fat on bread being preferred and missing answers can likely be interpreted as ”never” due to a misunderstanding of the structure of the question.

Many of the food groups where only moderate correlations were found between FFQ2020 and the reference method are foods generally not consumed daily, for example seafood and sugar-sweetened beverages. It is likely that more than four to six 24HDRs would have been needed to obtain a correct estimate by the reference method and this could have contributed to only moderate correlations for these food groups. Thus, to assess the validity of the FFQ-recorded food groups with moderate correlations, and conclude if those groups should be interpreted more cautiously in studies than others, they need to be compared with reference data covering a longer period.

During the study period from spring 2022 until autumn 2023, major events such as the covid-19 pandemic and increased prices due to the war in Ukraine have affected the civil society [36, 37]. This may have contributed to changes in food habits. Even so, the validity and reproducibility of FFQ2020 was found to be moderate to good.

Large epidemiological studies have to rely on cost-effective and easily administered tools for collection of lifestyle information. Hence, FFQs will always be relevant in studies of associations between long-term dietary intake and health. Our validation study shows that FFQ2020 can be used to capture intake of energy and nutrients, foods and food intake patterns among groups of people, with acceptable validity and reliability. The inclusion also of foods, food groups and food intake patterns are important, as dietary guidelines often target food intake rather than nutrient intake and it is food intake that may be affected by interventions in the population. Hence, research on associations between food intake patterns and health is crucial and for this purpose validation studies should also include diet intake information on this level.

The strengths of the present study relate to that the main part of the study group was recruited from the population-based MONICA project and that 93% of the participants in the validation group (*n* = 244) had completed six 24HDR recordings. Also, Spearman correlation coefficients were used as intakes of foods generally are skewed. Further, we evaluated relative validity not only for energy and nutrients but also for foods, food groups, and food intake patterns. However, there are limitations that should be acknowledged when the results are interpreted. First, we cannot exclude a selection bias effect since the fraction of eligible MONICA participants who gave final consent and completed the 24HDR recordings was low (24%), and people committed to a healthy lifestyle were overrepresented among those who were enrolled compared to the drop-out group. It should also be recognized that the allocation of foods and estimation of portion sizes used for weighting in energy and nutrient assessment are due to errors. However, these errors are likely random, with effects on the individual assessments but with less impact at the group level. Further, misreporting, primarily in the form of under-reporting, is a significant concern in self-reported dietary records, and it systematically correlates with factors such as age, sex, and BMI. Various strategies have been proposed to address this issue. In our study, we evaluated the estimated energy intake against the expected energy need and adjusted for the estimated energy intake. We discovered that underreporting distorts measures of energy and macronutrient intakes compared to vitamins and minerals. However, no method provides a complete correction. The decision on how to handle misreporting or underreporting rests with the researcher, who must consider the risk of misleading results and the potential loss of statistical power if observations are excluded. Finally, though the study was among the larger studies validating an FFQ against repeated 24HDRs, the power was not sufficient to stratify the analyses by gender, BMI or age group for the entire set of evaluated variables.

In conclusion, the results from the present study support FFQ2020 as a suitable instrument to estimate diet intake at the group level in large studies. Based on the results, it is recommended that participants lacking more than 10% of the food questions and/or a portion size indication as well as those with low or high energy intake in relation to their energy requirements are excluded in case-control or cohort studies.

### Electronic supplementary material

Below is the link to the electronic supplementary material.

Supplementary material file 1: **Table S1**: List of 30 surveyed food groups and components included in each group. **Table S2**: Description of the evaluated diet indices and included components. The symbol “(+)” denotes healthy food groups, with increasing scores assigned to higher rank classes. The symbol “(-)” indicates inverse scoring where higher ranks receive lower scores. **Table S3**: Median daily intakes with 25 and 75 percentile limits in 30 food groups as estimated from the FFQ or the 24HDR in the validation group (*n* = 244) together with Spearman correlation coefficients and ratios between intakes assessed by the two recording methods. **Table S4**: Proportion (%) classified to food group tertiles (1, 2, 3) based on ranking of increasing intakes by the FFQ2020 (test method) and 24HDR (reference) recordings among the 244 participants with at least four 24HDR recordings. For food groups indicated in bold, italic text more than 50% of the participants had not eaten the product in any of the recorded 24HDR days. **Table S5**: Daily mean (sd) intakes of energy and 14 nutrients based on FFQ2020 and 24hDR recordings in the validation group (*n* = 244) and after exclusion of the lowest 5% and highest 1% based on PAL values (*n* = 231) together with Pearson and Spearman correlation coefficients for absolute and energy-adjusted (residual and energy density methods) data. **Table S6**: Proportion (%) of energy and nutrients in tertiles (1, 2, 3) based on a ranking of increasing intakes by the FFQ2020 and 24HDR recordings, respectively. **Table S7**: Central measures (variations) of food index scores and intake in the 30 surveyed foods groups and the repeatability of the estimated intakes as assessed by intraclass correlation coefficient with 95% CI using a two-way mixed-effects model, absolute agreement, and average measures. **Table S8**: Mean (sd) daily intake of energy and 14 nutrients as estimated from the baseline FFQ2020 and the follow-up FFQ2020, Pearson correlation coefficients, and intraclass correlation coefficient with 95% CI using a two-way mixed-effects model, absolute agreement, and average measures. **Table S9**: Spearman correlation coefficients in 30 food groups and 3 diet indexes as estimated from the FFQ or the 24HDR in the validation group for men (*n* = 95) and women (*n* = 149), respectively. **Figure S1**: Scatter plot for (A) PAL values estimated from 24HDRs versus FFQ2020 (the dotted lines represent lower cut-off, i.e., PAL 0.7), and (B) daily energy intake estimated from 24HDRs versus FFQ2020

## Data Availability

According to the General Data Protection Regulation (GDPR), participant-level information cannot be made publicly available. Anonymized data are available after a reasonable request, appropriate ethical approval, and approval by the steering group at the Biobank Research Unit and the Northern Sweden Diet Database at Umeå University, Sweden (https://www.umu.se/en/biobank-research-unit/).

## Abbreviations

FFQ: Food Frequency Questionnaire
24HDR: 24-Hour Dietary Recall
NSDD: Northern Sweden Diet Database
VIP: Västerbotten Intervention Programme
MONICA: Monitoring Trends and Determinants in Cardiovascular Disease
rMED: Relative Mediterranean Diet Index
HNFI: Healthy Nordic Food Index
PDI: Plant-based Diet Index
PAL: Physical Activity Level
ICC: Intraclass Correlation Coefficient
BMI: Body Mass Index

## References

[1] Afshin A, Sur PJ, Fay KA, Ferrara G, Salama JS, Mullany EC et al. Health effects of dietary risks in 195 countries, 1990–2017: a systematic analysis for the global burden of Disease Study 2017. The Lancet (British edition). 2019;393(10184):1958–72. 10.1016/S0140-6736(19)30041-8 PMC6899507 30954305

[2] Willett W. Nutritional Epidemiology. 3rd ed. Oxford Academic. 2013.

[3] Johansson I, Hallmans G, Wikman A, Biessy C, Riboli E, Kaaks R. Validation and calibration of food-frequency questionnaire measurements in the Northern Sweden Health and Disease cohort. Public Health Nutr. 2002;5(3):487–96. 12003662 10.1079/PHN2001315

[4] Wennberg M, Vessby B, Johansson I. Evaluation of relative intake of fatty acids according to the Northern Sweden FFQ with fatty acid levels in erythrocyte membranes as biomarkers. Public Health Nutr. 2009;12(9):1477–84. 19144238 10.1017/S1368980008004503

[5] Johansson I, Guelpen BV, Hultdin J, Johansson M, Hallmans G, Stattin P. Validity of food frequency questionnaire estimated intakes of folate and other B vitamins in a region without folic acid fortification. Eur J Clin Nutr. 2010;64(8):905–13. 20502473 10.1038/ejcn.2010.80

[6] Klingberg S, Winkvist A, Hallmans G, Johansson I. Evaluation of plant sterol intake estimated with the Northern Sweden FFQ. Public Health Nutr. 2013;16(3):460–7. 22874465 10.1017/S1368980012003151 PMC10271774

[7] Johansson I, Nilsson LM, Stegmayr B, Boman K, Hallmans G, Winkvist A. Associations among 25-year trends in diet, cholesterol and BMI from 140,000 observations in men and women in Northern Sweden. Nutr J. 2012;11(1):40. 22686621 10.1186/1475-2891-11-40 PMC3489616

[8] Huseinovic E, Hörnell A, Johansson I, Esberg A, Lindahl B, Winkvist A. Changes in food intake patterns during 2000–2007 and 2008–2016 in the population-based Northern Sweden Diet Database. Nutr J. 2019;18(1):36. 31299991 10.1186/s12937-019-0464-0 PMC6626352

[9] Winkvist A, Klingberg S, Nilsson LM, Wennberg M, Renstrom F, Hallmans G, et al. Longitudinal 10-year changes in dietary intake and associations with cardio-metabolic risk factors in the Northern Sweden Health and Disease Study. Nutr J. 2017;16(1):20. 28351404 10.1186/s12937-017-0241-x PMC5370464

[10] Sikorski C, Yang S, Stennett R, Miller V, Teo 4, Anand SS, Paré G, Yusuf S, Dehghan M, Mente A. Changes in energy, macronutrient, and food consumption in 47 countries over the last 70 years (1950–2019): a systematic review and meta-analysis. Nutrition. 2023;108:111941. 36702047 10.1016/j.nut.2022.111941

[11] Santeramo FG, Carlucci D, De Devitiis, Seccia A, Stasi A, Viscecchia R, Nardone G. Emerging trends in European food, diets and food industry. Food Res Int. 2017;104:39–47. 29433781 10.1016/j.foodres.2017.10.039

[12] Eriksson M, Forslund A-S, Jansson J-H, Söderberg S, Wennberg M, Eliasson M. Greater decreases in cholesterol levels among individuals with high cardiovascular risk than among the general population: the northern Sweden MONICA study 1994 to 2014. Eur Heart J. 2016;37(25):1985–92. 26941200 10.1093/eurheartj/ehw052 PMC4929376

[13] Harris PA, Taylor R, Minor BL, Elliott V, Fernandez M, O’Neal L, et al. The REDCap consortium: building an international community of software platform partners. J Biomed Inf. 2019;95:103208. 10.1016/j.jbi.2019.103208 PMC7254481 31078660

[14] Lindroos AK, Petrelius Sipinen J, Axelsson C, Nyberg G, Landberg R, Leanderson P, et al. Use of a web-based Dietary Assessment Tool (RiksmatenFlex) in Swedish adolescents: comparison and validation study. J Med Internet Res. 2019;21(10):e12572. 31588902 10.2196/12572 PMC6914230

[15] Buckland G, Travier N, Cottet V, Gonzalez CA, Lujan-Barroso L, Agudo A, et al. Adherence to the mediterranean diet and risk of breast cancer in the European prospective investigation into cancer and nutrition cohort study. Int J Cancer. 2013;132(12):2918–27. 23180513 10.1002/ijc.27958

[16] Olsen A, Egeberg R, Halkjaer J, Christensen J, Overvad K, Tjonneland A. Healthy aspects of the Nordic diet are related to lower total mortality. J Nutr. 2011;141(4):639–44. 21346102 10.3945/jn.110.131375

[17] Satija A, Bhupathiraju SN, Rimm EB, Spiegelman D, Chiuve SE, Borgi L, et al. Plant-based dietary patterns and incidence of type 2 diabetes in US men and women: results from three prospective cohort studies. PLoS Med. 2016;13(6):e1002039. 27299701 10.1371/journal.pmed.1002039 PMC4907448

[18] Swedish Food Agency. Rapport 8 - Riksmaten - vuxna 2010-11 Livsmedels- och näringsintag bland vuxna i Sverige - metodrapport. https://www.livsmedelsverket.se/om-oss/psidata/apimatvanor2014. Accessed 13 Nov 2023.

[19] Mifflin MD, St Jeor ST, Hill LA, Scott BJ, Daugherty SA, Koh YO. A new predictive equation for resting energy expenditure in healthy individuals. Am J Clin Nutr. 1990;51(2):241–7. 2305711 10.1093/ajcn/51.2.241

[20] Willett WC, Howe GR, Kushi LH. Adjustment for total energy intake in epidemiologic studies. Am J Clin Nutr. 1997;65(4):S1220–8. 10.1093/ajcn/65.4.1220S 9094926

[21] Koo TK, Li MY. A Guideline of selecting and reporting Intraclass correlation coefficients for Reliability Research. J Chiropr Med. 2016;15(2):155–63. 27330520 10.1016/j.jcm.2016.02.012 PMC4913118

[22] Bland JM, Altman DG. Measuring agreement in method comparison studies. Stat Methods Med Res. 1999;8(2):135–60. 10501650 10.1177/096228029900800204

[23] Gu X, Wang DD, Sampson L, Barnett JB, Rimm EB, Stampfer MJ, et al. Validity and reproducibility of a Semiquantitative Food Frequency Questionnaire for Measuring Intakes of Foods and Food groups. Am J Epidemiol. 2024;193(1):170–9. 37552965 10.1093/aje/kwad170 PMC10773483

[24] Yuan C, Spiegelman D, Rimm EB, Rosner BA, Stampfer MJ, Barnett JB, et al. Validity of a Dietary Questionnaire assessed by comparison with multiple weighed Dietary records or 24-Hour recalls. Am J Epidemiol. 2017;185(7):570–84. 28338828 10.1093/aje/kww104 PMC5859994

[25] Yuan C, Spiegelman D, Rimm EB, Rosner BA, Stampfer MJ, Barnett JB, et al. Relative validity of nutrient intakes assessed by Questionnaire, 24-Hour recalls, and Diet Records as compared with urinary recovery and plasma concentration biomarkers: findings for women. Am J Epidemiol. 2018;187(5):1051–63. 29036411 10.1093/aje/kwx328 PMC5928456

[26] Al-Shaar L, Yuan C, Rosner B, Dean SB, Ivey KL, Clowry CM, et al. Reproducibility and validity of a semiquantitative food frequency questionnaire in men assessed by multiple methods. Am J Epidemiol. 2021;190(6):1122–32. 33350436 10.1093/aje/kwaa280 PMC8168140

[27] Troeschel AN, Hartman TJ, Flanders WD, Wang Y, Hodge RA, McCullough LE, et al. The American Cancer Society Cancer Prevention Study-3 FFQ has reasonable validity and reproducibility for Food groups and a Diet Quality score. J Nutr. 2020;150(6):1566–78. 32232407 10.1093/jn/nxaa082

[28] Sabir Z, Rosendahl-Riise H, Dierkes J, Dahl H, Hjartåker A. Comparison of dietary intake measured by a web-based FFQ and repeated 24-hour dietary recalls: the Hordaland Health Study. J Nutr Sci (Cambridge). 2022;11:e98–e. 10.1017/jns.2022.97 PMC9641502 36405094

[29] Rostgaard-Hansen AL, Rosthøj S, Brunius C, Olsen SF, Bjerregaard AA, Cade JE, et al. Relative validity and reproducibility of a web-based semi-quantitative food frequency questionnaire in the Danish Diet, Cancer, and Health—Next generations MAX Study. Nutrients. 2023;15(10):2389. 37242272 10.3390/nu15102389 PMC10222214

[30] Philippou E, Demetriou CA, Loucaides G, Solomonidou N, Critselis E, Polykarpou M, et al. Relative validity and reproducibility of the CyFFQ semiquantitative food frequency questionnaire for assessing dietary intake in Cypriot adults. J Hum Nutr Diet. 2023;36(1):139–53. 35567380 10.1111/jhn.13032 PMC10084044

[31] Imaeda N, Goto C, Sasakabe T, Mikami H, Oze I, Hosono A, et al. Reproducibility and validity of food group intake in a short food frequency questionnaire for the middle-aged Japanese population. Environ Health Prev Med. 2021;26(1):28–14. 33653279 10.1186/s12199-021-00951-3 PMC7923820

[32] Murakami K, Shinozaki N, Livingstone MBE, Kimoto N, Masayasu S, Sasaki S. Food combination questionnaire for Japanese: relative validity regarding food and nutrient intake and overall diet quality against the 4-day weighed dietary record. J Nutr Sci (Cambridge). 2023;12:e22–e. 10.1017/jns.2023.7 PMC9947634 36843967

[33] Huang Q, Zhou X, Zhang C, Huang L, Wang Q, Chen Q, et al. Relative validity and reproducibility of dietary measurements assessed by a semiquantitative food frequency questionnaire among Chinese healthy adults. Nutrients. 2023;15(3):545. 36771253 10.3390/nu15030545 PMC9920913

[34] Samson ML, Peeri NC, Alatise OI, O’Connell K, Sharma A, Ogunleye SG, et al. Validating a semi-quantitative food frequency questionnaire to assess regional diet in a study of cancer in South West Nigeria. Cancer Causes Control. 2023;34(6):495–503. 36995554 10.1007/s10552-023-01684-0 PMC10617682

[35] Abutbul Vered S, Shani Levi C, Rozen GA, Solt I, Rozen GS. Development and validation of a computerized web-based quantitative food frequency questionnaire. J Clin Nutr. 2022;52:169–77. 10.1016/j.clnesp.2022.10.012 36513450

[36] Mayasari NR, Ho DKN, Lundy DJ, Skalny AV, Tinkov AA, Teng IC, Wu MC, Faradina A, Mohammed AZM, Park JM, Ngu YJ, Aliné S, Shofia NM, Chang JS. Impacts of the COVID-19 pandemic on Food Security and Diet-related lifestyle behaviors: an Analytical Study of Google trends-based query volumes. Nutrients. 2020;12(10):3103. 33053656 10.3390/nu12103103 PMC7601866

[37] Laber M, Klimek P, Bruckner M, Yang L, Thurner S. Shock propagation from the Russia-Ukraine conflict on international multilayer food production network determines global food availability. Nat Food. 2023;4(6):508–17. 10.1038/s43016-023-00771-4 37322302 10.1038/s43016-023-00771-4

